# How a lipid mediates tumour suppression. Delivered on 29 June 2010 at the 35th FEBS Congress in Gothenburg, Sweden

**DOI:** 10.1111/j.1742-4658.2010.07900.x

**Published:** 2010-12

**Authors:** Harald Stenmark

**Affiliations:** 1 Centre for Cancer Biomedicine, Faculty of Medicine, University of OsloNorway; 2 Institute for Cancer Research, the Norwegian Radium Hospital, Oslo University HospitalMontebello, Norway

**Keywords:** autophagy, cancer, cell division, cytokinesis, endocytosis, PI 3-kinase, tumour suppressor

## Abstract

Phosphorylated derivatives of the membrane lipid phosphatidylinositol (PtdIns), known as phosphoinositides (PIs), regulate membrane-proximal cellular processes by recruiting specific protein effectors involved in cell signalling, membrane trafficking and cytoskeletal dynamics. Two PIs that are generated through the activities of distinct PI 3-kinases (PI3Ks) are of special interest in cancer research. PtdIns(3,4,5)*P*_3_, generated by class I PI3Ks, functions as tumour promotor by recruiting effectors involved in cell survival, proliferation, growth and motility. Conversely, there is evidence that PtdIns3*P*, generated by class III PI3K, functions in tumour suppression. Three subunits of the class III PI3K complex (Beclin 1, UVRAG and BIF-1) have been independently identified as tumour suppressors in mice and humans, and their mechanism of action in this context has been proposed to entail activation of autophagy, a catabolic pathway that is considered to mediate tumour suppression by scavenging damaged organelles that would otherwise cause DNA instability through the production of reactive oxygen species. Recent studies have revealed two additional functions of PtdIns3*P* that might contribute to its tumour suppressor activity. The first involves endosomal sorting and lysosomal downregulation of mitogenic receptors. The second involves regulation of cytokinesis, which is the final stage of cell division. Further elucidation of the mechanisms of tumour suppression mediated by class III PI3K and PtdIns3*P* will identify novel Achilles’ heels of the cell’s defence against tumourigenesis and will be useful in the search for prognostic and diagnostic biomarkers in cancer.

## Introduction

Eukaryotic cells contain very extensive intracellular membrane systems, and many vital cellular processes, such as metabolic reactions, signal transduction, cytoskeletal rearrangements, protein sorting and regulation of membrane dynamics, occur partially or entirely at membrane–cytosol interfaces. The main advantages of executing biochemical reactions on membranes include the limitation of substrate diffusion (i.e. limited to two dimensions instead of three) and the possibility of confining biochemical processes to restricted subcellular locations.

The containment of cellular processes to intracellular membranes requires the reversible assembly of protein complexes onto specific membranes. A group of phosphorylated derivatives of phosphatidylinositol (PtdIns), collectively known as phosphoinositides (PIs), are ideally suited for this task [1]. The PIs are generated and metabolized through the activities of a number of substrate-specific PI kinases and phosphatases, the dysfunctions of which are associated with various human diseases [2]. Of special interest in cancer research are two PI 3-kinases (i.e. kinases that phosphorylate the inositol headgroup in the 3-position) that have opposing roles in tumourigenesis. Class I PI 3-kinase (PI3K-I), on the one hand, is a well-known tumour promoting enzyme (or to be more precise, a small group of related enzymes) whose hyperactivity is strongly associated with carcinogenesis in humans [3]. Consistent with this, PTEN, a phosphatase that essentially reverses the 3-phosphorylation mediated by PI3K-I, is an important tumour suppressor [4]. The catalytic product of PI3K-I, PtdIns(3,4,5)*P*_3_, recruits several proteins involved in cell signalling to the plasma membrane, the most important one being the protein kinase AKT, which orchestrates various signalling cascades that promote cell growth and survival. On the other hand, class III PI 3-kinase (PI3K-III) is considered to be a tumour suppressor based on the findings that three of its accessory subunits, Beclin 1, UVRAG and BIF-1, have been independently identified as tumour suppressors whose partial or complete inactivation causes the increased occurrence of spontaneous tumours in mice and (probably) humans [5–7]. Recently identified molecular and cellular mechanisms that may serve to explain the tumour suppressor functions of PI3K-III are the topic of the present review.

## PI3K-III: a conserved lipid kinase complex

The catalytic subunit of PI3K-III was first identified as Vps34, an enzyme that mediates vacuolar protein sorting in the yeast *Saccharomyces cerevisiae* [8]. By contrast to PI3K-I, PI3K-III is conserved between yeast, plants and humans, and the human homologue of Vps34 is referred to as hVps34, PIK3C3 or VPS34. In the present review, the latter term is used. Subsequent work soon revealed that Vps34 is associated with a regulatory subunit, Vps15, a putative protein kinase [9]. More recently, Vps34 was found to engage in two functionally distinct complexes in yeast. One complex, consisting of Vps34, Vps15, Vps30 and Vps38, regulates endosomal sorting, whereas another complex, in which Vps38 is replaced by Atg14, is required for autophagy [10].

**Fig. 1:**
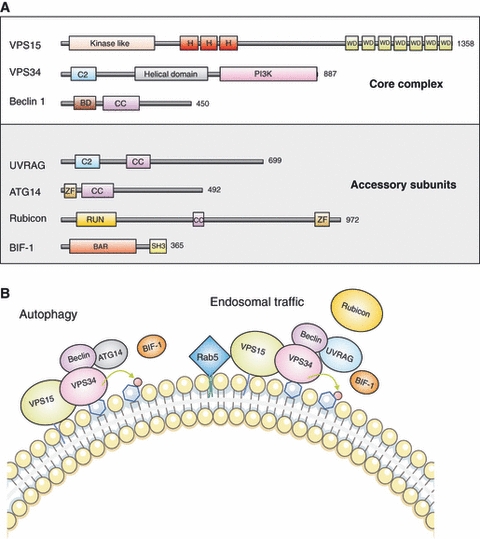
The human PI3K-III complex. (A) The various subunits of PI3K-III are indicated. VPS34, VPS15 and Beclin 1 are considered to form a core complex onto which accessory subunits can assemble. HEAT repeats (H), WD40 repeats (WD), Bcl-2 binding domain (BD), zinc fingers (ZF) and coiled-coil domains (CC) are indicated. Note that there exist alternative sequence variants for most of the subunits (not indicated). (B) Distinct PI3K-III subcomplexes regulate autophagosome formation and endosomal traffic.

The two PI3K-III complexes in yeast have their human counterparts: one consisting of VPS34, VPS15, the Vps30 homologue Beclin 1 and the Vps38 homologue UVRAG, and the other containing ATG14 (also called hAtg14, Atg14L or Barkor) instead of UVRAG [11–14]. Thus, we can consider VPS34-VPS15-Beclin 1 as a core complex onto which the accessory subunits UVRAG and ATG14 can assemble in a competitive manner (Fig. 1A). In addition, the UVRAG containing complex can associate with Rubicon, a protein that negatively regulates the function of this complex in endosomal and autophagic trafficking [11,12], and with BIF-1 (also known as endophilin B1 or SH3GLB1), identified as a positive regulator of autophagy [5] (Fig. 1B). Although Rubicon can be isolated as a major constituent of UVRAG-containing PI3K-III complexes, this is not the case with BIF-1, which appears to be more transiently associated [12,15]. Nevertheless, the fact that BIF-1 contains an amino-terminal BAR domain predicted to have membrane-bending ability, as well as the finding that knockdown of BIF-1 inhibits the catalytic activity of PI3K-III, suggests that this protein is an important accessory subunit of mammalian PI3K-III [5]. BAR domain proteins have been found to associate with membranes of high curvature [16], and it is tempting to speculate that BIF-1 might direct PI3K-III activity to such membranes.

The recently solved crystal structure of *Drosophila melanogaster* Vps34 revealed that this PI3K has a considerably smaller ATP-binding pocket than class I PI3Ks [17]. This explains why several inhibitors of class I PI3Ks fail to inhibit class III PI3K (i.e. they are too bulky to fit into the ATP-binding pocket). More importantly, the structural insight offers a rationale for designing specific PI3K-III inhibitors in the future.

Even though considerable knowledge has been gained about the biochemical composition of PI3K-III, we still know little about the regulation of its catalytic activity. The catalytic activity appears to be stimulated by BIF-1, whereas Rubicon inhibits the overall function of PI3K-III [5,11,12]. However, the exact mechanisms of these regulations remain unsolved. An important clue to the regulation of VPS34 has emerged recently with the finding that VPS34 is phosphorylated on threonine 159 by the cyclin-dependent kinase Ckd1 during mitosis, and that this causes its dissociation from Beclin 1 and an inhibition of autophagy [18].

## Recognition of PtdIns3*P* by FYVE and phox homology (PX) domains

A breakthrough in our understanding of how PI3K-III and its catalytic product control cellular functions came with the identification of the FYVE (conserved in Fab1, YOTB, Vac1 and EEA1) domain, and the demonstration that this domain binds PtdIns3*P*. The FYVE domain was originally identified as a zinc finger required for localization of the early endosomal autoantigen 1 (EEA1) to early endosomes [19]. The finding that wortmannin, a general PI3K inhibitor, prevents the localization of EEA1 to endosomes, provided a clue that EEA1 might be recruited by a 3-phosphorylated PI [20], and biochemical studies showed that the FYVE domains from yeast and mammalian proteins bind to PtdIns3*P* with high specificity [21–23]. Further progress in deciphering the downstream functions of PtdIns3*P* was obtained when the PX domain was identified as a second PtdIns3*P* binding domain [24–27]. Although a few mammalian PX domains bind to other 3-PIs than PtdIns3*P*, most of the PX domains bind specifically to PtdIns3*P* with affinities comparable to those of FYVE domains [28]. The human genome encodes approximately 30 FYVE domain-containing proteins and some 45 PX domain-containing proteins that presumably mediate most (but not all) of the downstream functions of PtdIns3*P* [29]. Additional PtdIns3*P*-binding proteins that do not contain FYVE or PX domains include the Proppin/WIPI proteins, which bind PtdIns3*P* [and to some extent the related PtdIns(3,5)*P*_2_] through a WD40-repeat-containing β-propeller structure [30,31], and certain variant pleckstrin homology domains such as the GLUE (GRAM-like ubiquitin-binding in EAP45) domain [32–34].

## Intracellular localization of PtdIns3*P*

The identification of PtdIns3*P*-binding domains offered the possibility to design probes that reveal the intracellular distribution of this lipid. Early work revealed that a single FYVE domain from the endosomal protein HRS binds PtdIns3*P* with too low affinity to be useful as a probe. Remarkably, however, when two FYVE domains from HRS were fused in tandem (2xFYVE), the resulting construct could be used for imaging of cellular PtdIns3*P* with high sensitivity and specificity, presumably as a result of an avidity effect [35]. The fact that 2xFYVE can be either transfected into cells as a fusion with green fluorescent protein or another tag, or expressed in bacteria and purified as a recombinant probe that can be used directly on fixed specimens, makes this probe very versatile for monitoring the distribution of PtdIns3*P*. Subsequently, other probes have been used for monitoring PtdIns3*P*, including the FYVE domain of SARA, which has an intrinsic ability to dimerize and therefore does not need to be expressed as tandem fusion [36], and certain PX domains [37]. In general, the various probes have yielded comparable results, although the 2xFYVE probe has been most rigorously tested for ligand specificity. The original studies using 2xFYVE by fluorescence and electron microscopy showed that the bulk of PtdIns3*P* is associated with the limiting and intraluminal membranes of endosomes at steady-state [35]. Subsequent studies have revealed that PtdIns3*P* can also be detected at the plasma membrane of cells stimulated with insulin or lysophosphatidic acid [38,39]. Because this pool of PtdIns3*P* is generated through the activity of PI3K-II [40], which has so far not been implicated in tumour suppression, it will not be considered further in the present review. Recent analyses of starved yeast cells have revealed a strong localization of PtdIns3*P* on autophagosomes, especially on the inner membranes [41], and, during induction of autophagy in mammalian cells, PtdIns3*P* is formed on membranes of the endoplasmic reticulum (ER) [42]. The importance of this is discussed below.

## PI3K-III and PtdIns3*P* binding proteins in endosomal trafficking

**Fig. 2:**
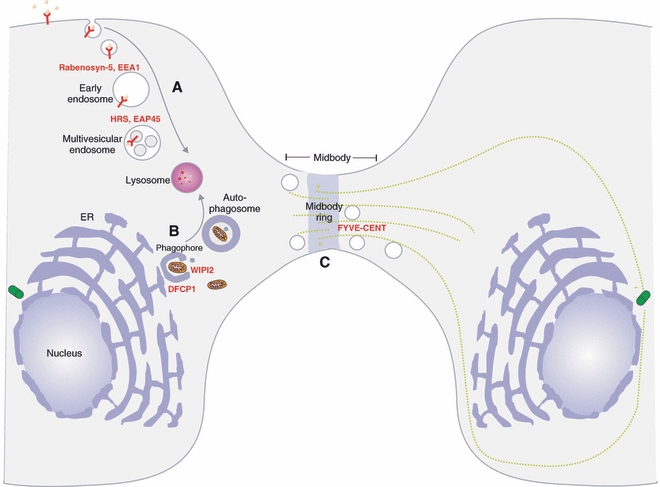
PtdIns3*P* effectors in cell regulation. Endocytic downregulation of a mitogenic receptor (A), autophagy (B) and cytokinesis (C). PtdIns3*P* effectors involved in the various processes are shown in red. Rabenosyn-5 and EEA1 mediate membrane fusion in the early endocytic pathway. HRS and EAP45 are subunits of the ESCRT machinery that sorts ubiquitinated mitogenic receptors into the ILVs of multivesicular endosomes. DFCP1 mediates phagophore biogenesis, whereas WIPI2 mediates autophagosome biogenesis. FYVE-CENT facilitates cytokinesis. Microtubules are indicated by dashed green lines.

Because Vps34 and Vps15 were originally identified as mediators of vacular protein sorting in yeast, the first characterized functions of PI3K-III and PtdIns3*P* were those associated with endosomal trafficking (Fig. 2A).

### PtdIns3*P* and endosomal fusion

Yeast cells devoid of functional Vps34 or Vps15 secrete several hydrolases that normally are transported to the lysosome-like vacuole [8,43], suggesting that these proteins control trafficking between the secretory and endosomal pathways and/or between endosomes. A key effector of PtdIns3*P* in endocytic trafficking is the FYVE-domain-containing protein Vac1, which interacts genetically and physically with the small GTPase Vps21, and with Vps45, a member of the Sec1/Munc18 family of proteins regulating the formation of SNARE complexes that mediate membrane fusion [44,45]. Indeed, studies of the mammalian homologues of Vac1, Vps21 and Vps45, termed Rabenosyn-5, RAB5 and VPS45, respectively, have revealed that these proteins function in tethering and fusion reactions in the early endocytic pathway [46]. Moreover, studies in *Drosophila* have revealed that Rabenosyn functions to bridge Rab5 with Vps45, thereby regulating the function of the SNARE protein Avalanche in endosomal fusion [47]. Interestingly, functional interference with Rabenosyn-5 and its interacting partners causes a loss of both epithelial and planar polarity [47,48]. The loss of epithelial polarity is a prevailing characteristic of carcinomas, and mutation of Rabenosyn indeed causes epithelial tumours in *Drosophila*. To date, the mechanisms by which Rabenosyn controls epithelial polarity have not been elucidated, whereas more detailed insight is available in the case of planar cell polarity. One consequence of interferring with Rabenosyn function is that Flamingo, a determinant of planar cell polarity through the noncanonical Wnt signalling pathway, accumulates in the cytoplasm instead of translocating to polarized membrane domains [48]. Accumulating evidence suggests a link between improper development of planar cell polarity and cancer [49], and even though it is still not clear whether Rabenosyn-5 is a tumour suppressor in mammals, the epithelial and planar cell polarity maintained by this RAB5 and PtdIns3*P* effector has to be considered when dissecting the tumour suppressor activities of PI3K-III.

Early studies in mammalian cells have also revealed another important PtdIns3*P* effector in endosomal tethering and fusion, namely EEA1, a protein that contains a Rab5 binding domain and a FYVE domain at its C-terminus, and a distinct Rab5 binding domain at its N-terminus [50]. EEA1 forms rod-shaped dimers through parallel coiled-coil interactions, and is well suited for tethering two opposing Rab5-containing membranes [51]. The exquisite localization of EEA1 to early endosomes is probably conferred by the coincident detection of PtdIns3*P* and GTP-bound Rab5 [50]. EEA1 is structurally related to Rabenosyn-5, and also interacts with SNARE molecules [52,53]. In a remarkable reconstitution of Rab5-mediated fusion using liposomes and recombinant SNAREs and Rab5 effectors, the inclusion of PtdIns3*P* could bypass the requirement for PI3K-III, as expected based on previous studies [54]. Importantly, the omission of either EEA1 or Rabenosyn-5 was sufficient to inhibit fusion strongly, indicating that these proteins, despite their structural relatedness, have nonredundant functions in endocytic membrane fusion.

### PtdIns3*P* and endosomal sorting to the degradative pathway

Consistent with the fact that Vps34 was originally identified as a mediator of protein sorting, studies of both yeast and mammalian cells have shown that PI3K-III is required for proper sorting of certain membrane proteins from endosomes to vacuoles/lysosomes [8,55]. Moreover, interference with the function of VPS34 in mammalian cells results in dilated late endosomes devoid of intraluminal vesicles (ILVs) [56,57]. Recent studies have revealed that not only VPS34, but also VPS15, Beclin 1, UVRAG and BIF-1 are required for proper degradation of endocytosed epidermal growth factor receptors in lysosomes, highlighting the involvement of an entire PI3K-III complex in endosomal sorting [15].

A mechanistic explanation for these findings has emerged with the discovery of the endosomal sorting complex required for transport (ESCRT) machinery [58,59]. This molecular machinery, which consists of at least four subcomplexes (ESCRT-0, -I, -II and -III), is recruited to endosome membranes where it recognizes ubiquitinated membrane proteins (e.g. activated growth factor receptors and membrane-anchored hydrolases) and sorts these into ILVs. Recent reconstitution studies employing giant unilamellar vesicles have revealed that ESCRT-0, which contains as many as five ubiquitin-binding domains, serves to sequester ubiquitinated cargoes, whereas ESCRT-I and -II, which also contain ubiquitin-binding domains, serve to form invaginations of the endosomal membrane [60]. Finally, ESCRT-III is recruited, cargo is deubiquitinated by deubiquitinating enzymes recruited by ESCRT-III [61], and ESCRT-III serves to sever the neck of the forming invagination, thereby securing the inclusion of cargo proteins within ILVs [60]. The main link between PI3K-III and the ESCRT pathway is the fact that Vps27/HRS, a core component of ESCRT-0, contains a FYVE domain that mediates its recruitment to endosomal membranes through binding PtdIns3*P* [62]. Vps27/HRS in turn recruits ESCRT-I through interaction with the Vps23/TSG101 subunit, so the initial recruitment of ESCRT-0 to endosomal membranes via FYVE-PtdIns3*P* interactions is crucial for the function of the ESCRT machinery. In addition, a subunit of ESCRT-II, Vps36/EAP45, contains a PtdIns3*P*-binding GLUE domain that is predicted to contribute to the membrane recruitment of ESCRT-II [32,34]. The involvement of the ESCRT machinery in protein sorting and ILV biogenesis, as well as the notion that key subunits of this machinery require PtdIns3*P* for their membrane recruitment, readily explains why interference with PI3K-III functions results in impaired protein sorting and causes endosomes devoid of ILVs.

## PI3K-III and PtdIns3*P* binding proteins in autophagy

Macroautophagy (referred to here as autophagy) is a process that involves the sequestration of cytoplasm by a double membrane called phagophore or isolation membrane, followed by fusion of the resulting autophagosome with endosomes and lysosomes [63] (Fig. 2B). The degradation of the sequestered cytosolic material in autolysosomes is beneficial for the cell, both under starvation conditions (when it is crucial to recycle free amino acids by degrading cytosolic proteins that are not housekeeping), during infection with cytosolic parasites, and under various stress conditions (e.g. those that result in the accumulation of cytosolic protein aggregates that are not readily degraded by proteasomes). The exact origin of the phagophore membrane is still a matter of debate, although there are strong arguments in favour of a contribution from both ER and mitochondrial membranes [42,64,65]. At least in the case of ER membranes, there is evidence for the production of PtdIns3*P* during the early phase of autophagosome formation [42,66]. Several lines of evidence point to a crucial role for PI3K-III in autophagy [66], and immunoelectron microscopy of starved yeast cells using the 2xFYVE probe has revealed that PtdIns3*P* is enriched on inner side of the phagophore during autophagosome formation [41].

Studies in yeast have revealed that a complex consisting of Vps34, Vps15, Vps30 and Atg14 is required for autophagy [10], and subsequent work in mammalian cells has shown a similar requirement for the mammalian homologues of these proteins [11,12]. In addition, an involvement of the Vps38 homologue UVRAG has been reported [7], which is surprising in light of the strong evidence that Vps38 mediates endosomal trafficking and not autophagy in yeast. A possible role of UVRAG in autophagy is supported by the independent identification of BIF-1, an interactor of UVRAG, as a regulator of autophagy [5]. On the other hand, monoallelic UVRAG mutations associated with microsatellite unstable colon cancer have no effect on autophagy, and the depletion of UVRAG has an undetectable effect on autophagy in HEK293 cells, whereas endosomal sorting is affected [67]. One explanation for the conflicting findings on UVRAG may stem from the involvement of UVRAG in fusion between autophagosomes (and late endosomes) and lysosomes through its interactions with the HOPS complex [68]. Except for the catalytic activity of VPS34, little is known about the specific functions of the various PI3K-III subunits in autophagy. However, recent evidence suggests that the specific function of ATG14 in autophagy may reflect the ability of this protein to target PI3K-III to ER membranes [69].

How does PtdIns3*P* mediate autophagy? The only known PtdIns3*P* effector in autophagy in yeast is the Proppin protein Atg18 [70], whose binding to PtdIns3*P* is required for autophagy [71]. Although the exact function of Atg18 in autophagy remains to be clarified, current evidence suggests that this protein, in complex with Atg2, facilitates the recruitment of lipidated Atg8, a key effector in autophagosome formation to phagophore membranes [71]. A mammalian Atg18 homologue, WIPI2, is recruited to phagophore membranes along with ULK1, a protein kinase that positively regulates autophagy [72]. Interestingly, the depletion of WIPI2 leads to a strong accumulation of omegasomes, comprising ER-localized PtdIns3*P*-containing structures positive for DFCP1 (double FYVE domain-containing protein 1) that are considered to act as platforms for autophagosome formation. This suggests a role for WIPI2 in the progression from omegasomes into autophagosomes.

## PI3K-III and PtdIns3*P* binding proteins in cytokinesis

A surprising finding when using a green fluorescent protein-tagged version of the 2xFYVE probe was that PtdIns3*P* accumulates in the bridge separating two dividing cells, the so-called midbody. PtdIns3*P* is frequently associated with the central, electron-dense part of the midbody, referred to as the midbody ring or the Flemming body, but can also be observed on small vesicles throughout the midbody region [73]. This localization of PtdIns3*P* raises the question of whether its formation is required during cytokinesis, the final stage of cell division (Fig. 2C). Indeed, small interfering RNA-mediated knockdown of VPS34, as well as of the accessory PI3K-III components VPS15, Beclin 1, UVRAG and BIF-1, causes an increased proportion of cells undergoing cytokinesis, suggesting a role for PI3K-III in the completion of cytokinesis [15,73]. Small interfering RNA screening identified the large FYVE domain-containing protein FYVE-CENT (FYVE protein on centrosomes) as a PtdIns3*P* effector in cytokinesis. FYVE-CENT localizes to the centrosome in interphase cells and translocates to the midbody during telophase. This translocation appears to be mediated by the microtubule-based motor protein KIF13A. The precise function of FYVE-CENT during cytokinesis remains to be characterized, although one clue arises from the finding that TTC19, a protein that associates with FYVE-CENT and accompanies it on its translocation from the centrosome to the midbody, interacts with the ESCRT-III subunit CHMP4B [73]. This is interesting because ESCRT-III has been proposed to mediate the final membrane abscission step during cytokinesis [74,75], and it is possible that TTC19, brought to the midbody by FYVE-CENT and KIF13A, may be a positive regulator of CHMP4B in cytokinesis. By analogy with its yeast counterpart Vps32/Snf7, CHMP4B is predicted to form spiral-shaped oligomers that constrict to mediate membrane severing [76], and TTC19 might serve to control this oligomerization.

## PI3K-III and PtdIns3*P* effectors in tumour suppression

The PI3K-III subunit Beclin 1 is monoallelically deleted in a large proportion of breast and ovarian cancers, and heterozygous *beclin 1* knockout mice develop spontaneous mammary tumours [6]. These findings, combined with the observation that both Beclin 1 and its yeast homologue Vps30 mediate autophagy, suggest that Beclin 1 acts as a tumour suppressor because of its function in autophagy. In support of this idea, there is evidence that autophagy functions as tumour suppressor by scavenging damaged mitochondria and peroxisomes that would otherwise cause genomic instability through oxygen radical-induced DNA damage [77]. Further supporting the notion that PI3K-III acts as a tumour suppressor through its function in autophagy is the observation that two other PI3K-III accessory proteins identified as positive regulators of autophagy, UVRAG and BIF-1, are also tumour suppressors [5,7]. Even though these are compelling data, one obvious question arises regarding the role of PI3K-III in autophagy-mediated tumour suppression: are other autophagy regulators also tumour suppressors? One would expect that this would be the case but, so far, only one of the many other proteins implicated in autophagy regulation has been identified as a putative tumour suppressor, the protease ATG4C [78]. Because, among the more than 30 positive regulators of autophagy, three out of four identified tumour suppressors belong to the PI3K-III complex, the possibility exists that PI3K-III may mediate tumour suppression not by autophagy but by an alternative means.

Given the importance of endocytosis and lysosomal downregulation of growth factor receptors in attenuation of mitogenic cell signalling [79], one distinct possibility is that PI3K-III could (at least in part) exert its tumour suppressor function through its role in endosome fusion and endosomal receptor sorting. Although there is no direct evidence that this is the case, it is interesting to note that the PtdIns3*P*-binding endosomal fusion regulator, Rabenosyn, is a tumour suppressor in flies [47], and the same is the case with multiple components of the ESCRT machinery that acts downstream of PtdIns3*P* in the endosomal sorting of mitogenic receptors [80–83]. Arguing against this idea is the fact that Hrs, the PtdIns3*P* binding ESCRT-0 protein that initiates further ESCRT recruitment to membranes, is not a tumour suppressor in *Drosophila*.

The recent discovery that PI3K-III regulates cytokinesis has offered a third possible explanation for the tumour suppressor activity of this enzyme complex [73]. Inhibition of PI3K-III activity not only causes an increased proportion of cells undergoing cytokinesis, but also an increase in bi- and multinucleate cells. Under certain conditions, tetraploidy may develop into aneuploidy, which is strongly associated with cancer. It is therefore likely that incomplete cytokinesis, as experienced when PI3K-III or the PtdIns3*P* effector FYVE-CENT is functionally ablated, may represent a step in oncogenesis [84]. It is interesting to note that FYVE-CENT is frequently mutated in breast cancer [85,86], although it remains to be established whether this PtdIns3*P* effector is a genuine tumour suppressor.

## Conclusions and perspectives

**Fig. 3:**
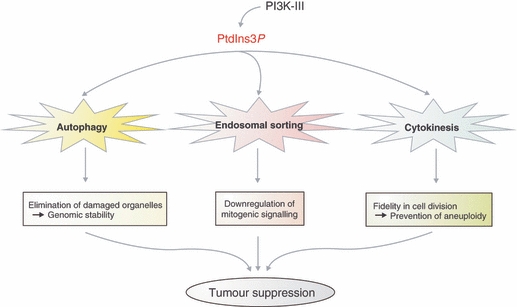
Alternative tumour suppressor mechanisms of PI3K-III. Three alternative (hypothetic) tumour suppressor mechanisms downstream of PtdIns3P formation are shown. The relative importance of these mechanisms in the tumour suppressor activity of PI3K-III subunits remains to be established.

As discussed in the present review, PI3K-III may theoretically function as a tumour suppressor via at least three different mechanisms (Fig. 3). The involvement of PI3K-III in autophagy maintains genome stability by eliminating damaged organelles that produce reactive oxygen species; the role of PI3K-III in endosomal fusion and sorting ensures correct downregulation of mitogenic signalling; and the correct function of PI3K-III in cytokinesis prevents aneuploidy. Further work is required to establish which of these three processes is most important in PI3K-III-mediated tumour suppression. It can also not be excluded that PI3K-III may act as tumour suppressor by additional means. For example, SARA, a mediator of transforming growth factor-β signalling, requires PtdIns3*P* binding for its function [87], and the transforming growth factor-β signalling pathway does have a tumour suppressor role under most conditions [88]. Furthermore, PtdIns3*P* binding subunits mediate membrane association of the retromer complex [89], which sorts cargoes such as mannose 6-phosphate receptors and Wntless (an accessory factor in Wnt secretion) for trafficking from endosomes to the biosynthetic pathway [89,90]. Even though there is no evidence so far that implicates the retromer in tumour suppression, this possibility cannot at present be discarded.

The involvement of PI3K-III in diverse cellular processes raises the question of how this complex is recruited to the correct membranes at the right time. There is evidence that PI3K-III is recruited to early and late endosomal membranes through interactions with the small GTPases RAB5 and RAB7, respectively [91–93]. Less is known about how PI3K-III is recruited to midbodies and autophagic membranes, although the latter is likely to be mediated by the autophagy-specific subunit ATG14 [69]. In addition, the finding that phosphorylation of VPS34 during mitosis causes its dissociation from Beclin 1 [18] provides a hint that post-translational modifications of PI3K-III may contribute to regulate its activity and specificity.

Although considerable efforts have been made to understand how PtdIns3*P* is formed by PI3K-III, we are also beginning to learn about the metabolism of this lipid. Three known routes for PtdIns3*P* metabolism have been described: degradation by lysosomal lipases, phosphorylation by the PtdIns3*P* 5-kinase Fab1/PIKfyve, and dephosphorylation by 3-phosphatases [94]. It is worth noting that Fab1/PIKfyve is itself a PtdIns3*P* binding protein [95], and that MTM1 and MTMR2, two phosphatases capable of dephosphorylating PtdIns3*P*, can associate with PI3K-III on endosomal membranes [96,97]. This suggests a tight regulation of PtdIns3*P* formation and turnover, and it will be interesting to determine whether PIKfyve and MTM1/MTMR2 may play any role in tumourigenesis.

Even though it is assumed that PI3K-III functions as tumour suppressor through its ability to produce PtdIns3*P* at the correct intracellular membranes, this has not been formally demonstrated and, to date, we do not know whether the catalytic subunit of PI3K-III, VPS34, is a tumour suppressor. Further studies should clarify this, and it will also be important to establish whether the catalytic function of VPS34 is required for its eventual tumour suppressor function.

If we nevertheless accept that PI3K-III acts as tumour suppressor through PtdIns3*P* generation, can this be exploited in cancer diagnosis and therapy? Because it is much easier to inhibit an enzyme pharmacologically than to boost its function, the tumour promotor PI3K-I is a more attractive drug target than the tumour suppressor PI3K-III. On the other hand, we know that PtdIns3*P* can be dephosphorylated and that PI3K-III undergoes negative regulation [11,12], and alleviation of these inhibitory mechanisms might provide a viable strategy towards increasing the tumour suppressor activity of PI3K-III and its catalytic product in cancer treatment. More straightforwardly, knowing that PI3K-III subunits such as Beclin 1 and UVRAG are frequently downregulated or mutated in cancers [6,7,67,98,99], it will be interesting to perform systematic analyses of PI3K-III subunits and key PtdIns3*P* effectors in various cancers. Such studies should reveal mutational and expression-based signatures that can be used to predict the outcome of disease, and to guide the choice of therapeutic regimens.

## Abbreviations

EEA1: early endosomal autoantigen 1
ER: endoplasmic reticulum
ESCRT: endosomal sorting complex required for transport
ILV: intraluminal vesicles
PI: phosphoinositide
PI3K: phosphoinositide 3-kinase
PtdIns: phosphatidylinositol
PX: phox homology
